# An unusual lead point: jejunal leiomyosarcoma causing jejuno-jejunal intussusception – a case report

**DOI:** 10.1097/RC9.0000000000001003

**Published:** 2026-09-14

**Authors:** Mayin Uddin Mahmud, Sanjoy Kumar Nath

**Affiliations:** Department of Surgery, Chittagong Medical College and Hospital, Chattogram, Bangladesh

**Keywords:** adult intussusception, case report, jejunal leiomyosarcoma, mesenchymal tumor, small bowel obstruction, smooth muscle tumor

## Abstract

**Introduction:**

Adult intussusception is rare. Unlike in pediatric cases, adult intussusception typically arises from an underlying pathology. Primary jejunal leiomyosarcoma is an exceptionally rare malignant tumor originating from the smooth muscle cells of the small intestine.

**Case presentation:**

A 52-year-old man presented with signs and symptoms of acute intestinal obstruction, including abdominal pain, constipation, vomiting, and abdominal distension of 2 days duration. Contrast-enhanced computed tomography revealed a jejunojejunal intussusception with a lead-point intraluminal mass. Exploratory laparotomy showed a jejuno-jejunal intussusception 40 cm distal to the duodenojejunal flexure. Segmental resection with primary anastomosis was performed. The postoperative period was uneventful. Histopathology demonstrated a spindle-cell neoplasm consistent with leiomyosarcoma. Immunohistochemistry showed strong positivity for smooth muscle actin and desmin, and negativity for CD117, DOG-1, and S-100.

**Discussion:**

Primary jejunal leiomyosarcomas often present with nonspecific symptoms. Imaging, especially CT, is critical for diagnosis, but confirmation requires histopathology and immunohistochemistry. Complete surgical resection remains the treatment of choice. This case contributes a complete immunohistochemical panel that distinguishes leiomyosarcoma from gastrointestinal stromal tumors, the formal AJCC 8th edition staging, and a rationale for adjuvant chemotherapy based on soft tissue sarcoma protocols – thereby providing a more standardized diagnostic and management template than existing jejunal leiomyosarcoma case reports.

**Conclusion:**

Jejunal leiomyosarcoma presenting as jejuno-jejunal intussusception is exceptionally rare. Early recognition and prompt surgical intervention are essential for favorable outcomes.

## Introduction

Adult intussusception is an uncommon surgical condition, accounting for approximately 5% of all intussusceptions and 1–5% of all intestinal obstructions^[^1,2^]^. Pediatric intussusception is usually idiopathic, whereas adult intussusception typically occurs secondary to a structural lesion, such as a neoplasm, Meckel’s diverticulum, or inflammatory polyp^[^3^]^.

The small intestine comprises approximately two-thirds of the gastrointestinal tract (GIT); however, the incidence of malignancies in this part is less than 5% of total GIT malignancies^[^4^]^. Carcinoids are the most common small intestine neoplasms (44.3%), followed by adenocarcinomas (32.6%), lymphomas (14.7%), gastrointestinal stromal tumors (GISTs) (7.2%), and leiomyosarcomas (LMS) (1.2%)^[^5–7^]^. Symptoms of LMS of the small intestine include GIT bleeding, nonspecific abdominal pain, weight loss, intestinal obstruction, and perforation^[^8–10^]^. In rare circumstances, it may present as the lead point (LP) of intussusception, as observed in the present case^[^11,12^]^. The diagnosis of jejunal neoplasms, including LMS, is challenging because of a nonspecific presentation, an uncommon location, and limitations of conventional endoscopic methods. Advanced imaging modalities, such as computed tomography and magnetic resonance imaging, are helpful, but histological examination is the main tool for accurate diagnosis^[^12^]^.

HIGHLIGHTS

- Jejunal leiomyosarcoma is rare but carries a poor prognosis.
- The jejunum is an unusual site for intussusception.
- Presentations are nonspecific; leiomyosarcoma serving as a lead point for intussusception is extremely rare.
- Complete resection is the mainstay of treatment.
- The role of adjuvant therapy remains a matter of significant debate due to the lack of standardized guidelines.

Despite advancements in gastrointestinal imaging, primary jejunal LMS remains an extremely rare clinical condition. The existing literature documents small intestinal malignancies such as GISTs and adenocarcinomas; however, primary jejunal LMS has been sparsely reported. Furthermore, while intussusception is a hallmark in pediatric populations, its occurrence in adults is rare. The presentation of a jejuno-jejunal LMS as the pathological LP of intussusception represents a “rare-on-rare” clinical presentation that is sparsely published in the surgical literature. The aim of this paper is to present a rare case of primary jejunal leiomyosarcoma presenting as jejuno-jejunal intussusception in a 52-year-old man, which was managed successfully with surgical resection, and to provide a comprehensive review of the relevant literature. By detailing the clinical, radiological, and histological findings, this report seeks to enhance diagnostic awareness of rare mesenchymal tumors as LPs in adult bowel obstructions and to emphasize the role of immunohistochemistry in distinguishing LMS from common neoplasms of the small intestine, such as carcinoids. This patient was diagnosed and managed in a tertiary academic hospital. This case report was prepared in accordance with the Surgical Case Report (SCARE) 2025 guidelines^[^13^]^.

## Case presentation

A 52-year-old male presented to the emergency department with complaints of diffuse abdominal pain, abdominal distension, constipation, and vomiting for 2 days. He had no history of prior abdominal surgery, hematemesis, melena, or weight loss. He had no family history of malignancy. Allergy and social histories were unremarkable. On examination, he was tachycardic but normotensive. His abdomen was markedly distended and tender in the central region, with hyperactive bowel sounds. Laboratory investigations revealed mild leukocytosis. A plain abdominal X-ray showed multiple air-fluid levels centrally. Contrast-enhanced computed tomography (CECT) of the abdomen and pelvis demonstrated a typical “target sign” consistent with jejuno-jejunal intussusception and a lead-point intraluminal soft-tissue mass measuring approximately 4.5 × 4 cm (Fig. 1). Carcinoembryonic antigen and cancer antigen 19-9 were normal. A provisional diagnosis of jejuno-jejunal intussusception due to a neoplastic cause was made. Differential diagnoses included GIST, adenocarcinoma, and leiomyosarcoma. Routine preoperative investigations were unremarkable. The patient was resuscitated with intravenous crystalloids to correct fluid and electrolyte imbalance; a nasogastric tube was placed for bowel decompression; a Foley catheter was inserted for monitoring urine output; and prophylactic broad-spectrum intravenous antibiotics (cefuroxime and metronidazole) were administered. Following optimization, the patient was taken for exploratory laparotomy.

**Figure 1.**
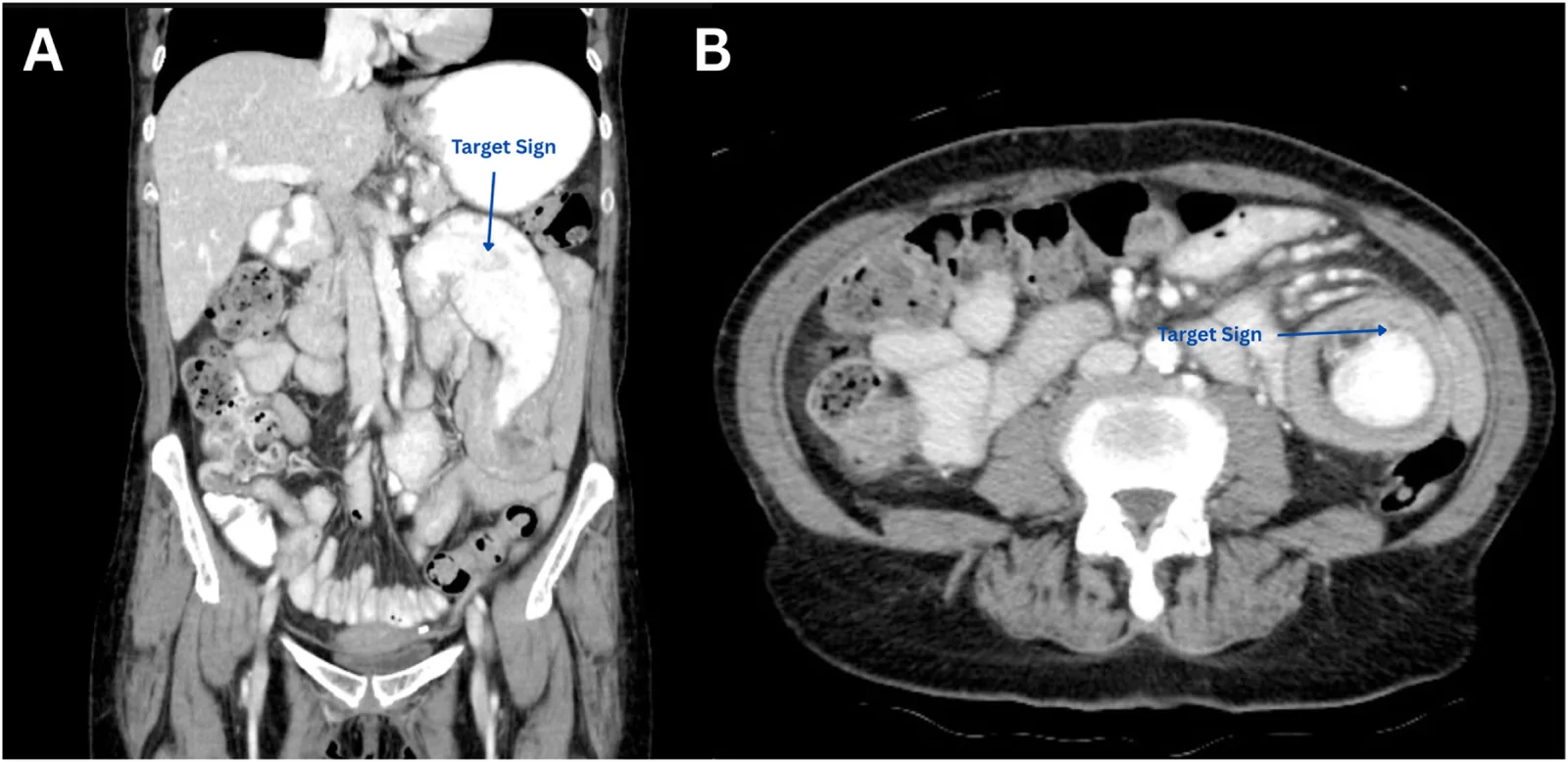
CT scan of the abdomen: (A) coronal image showing jejuno-jejunal intussusception with an intraluminal soft-tissue mass serving as a lead point (arrow); (B) axial image showing a “target sign” (arrow). CT, computed tomography.

Exploratory laparotomy was performed under general anesthesia with endotracheal intubation, and the patient was positioned supine. Standard skin preparation with povidone-iodine and draping was carried out, and the abdomen was entered via a midline incision. The procedure was performed by a consultant general surgeon with 15 years experience and revealed a jejuno-jejunal intussusception with a soft-tissue lesion as the LP located approximately 40 cm distal to the duodenojejunal flexure (Fig. 2). The serosa was not involved. No mesenteric, peritoneal, or liver metastases were noted. A segmental resection of the involved jejunal segment with 10 cm distal and proximal margins and mesenteric lymphadenectomy were performed without reduction of the intussusception according to oncological principles. End-to-end jejunal anastomosis was performed using a GIA stapler with the DST series (Medtronic). The skin-to-skin operative time was 2 hours.

**Figure 2.**
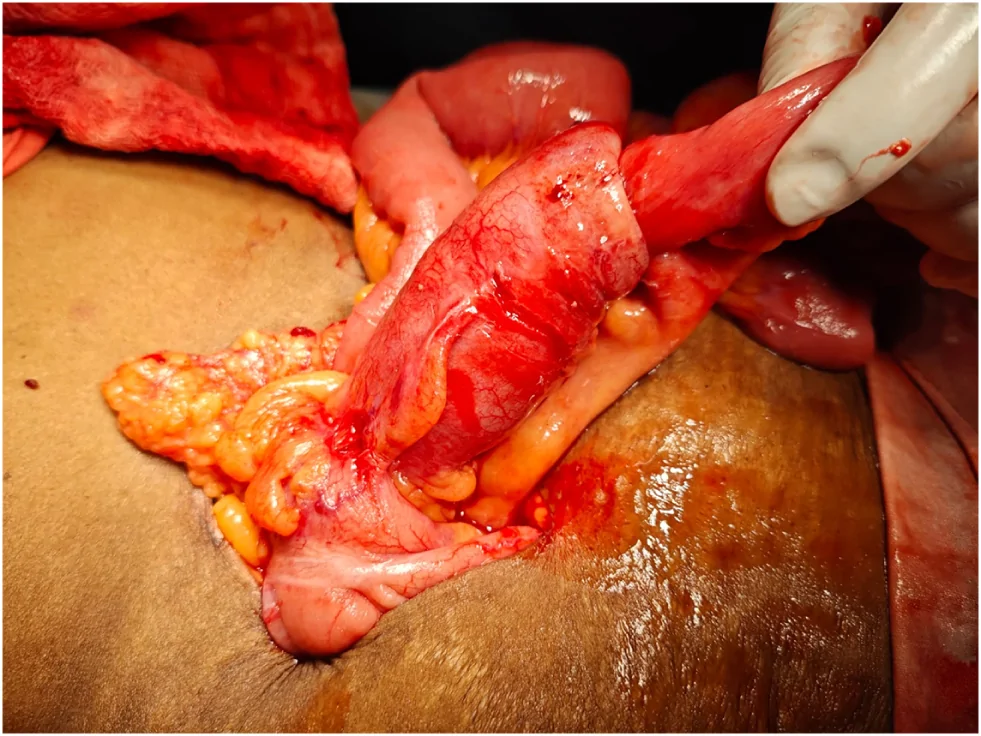
Intraoperative image showing jejuno-jejunal intussusception.

Gross examination showed an intraluminal polypoidal tumor measuring 4.5 × 4 × 3.5 cm with a smooth surface invaginating into the distal lumen. Microscopy revealed interlacing bundles of spindle cells with eosinophilic cytoplasm and elongated nuclei exhibiting mild-to-moderate atypia with areas of necrosis. Tumor grade was high. The Ki-67 index was 70–80%. Both margins were free of tumor, with a final pathological clearance of approximately 7 cm. The reduction from the intraoperative 10 cm surgical margin to the 7 cm pathological margin is attributable to specimen shrinkage following formalin fixation. Seven lymph nodes were identified, but no metastases were found in the lymph nodes. Lymphovascular and perineural invasion were absent. Pathological stage was pT2, pN0, and pMx (according to the AJCC soft tissue sarcoma staging)^[^14^]^. Mitotic figures were 4–5 per 10 high-power fields. Immunohistochemistry demonstrated strong positivity for smooth muscle actin (SMA) and Desmin, and negativity for CD117, DOG-1, and S-100 (Fig. 3). These findings confirmed the diagnosis of primary jejunal leiomyosarcoma. The postoperative period was uneventful, and the patient was discharged 7 days after the operation. The multidisciplinary tumor board initiated infusion therapy with cisplatin and doxorubicin. The patient tolerated the chemotherapy well and completed the scheduled regimen. The patient was followed up for 6 months in good condition. The chronological sequence of events is summarized in Table 1.

**Figure 3.**
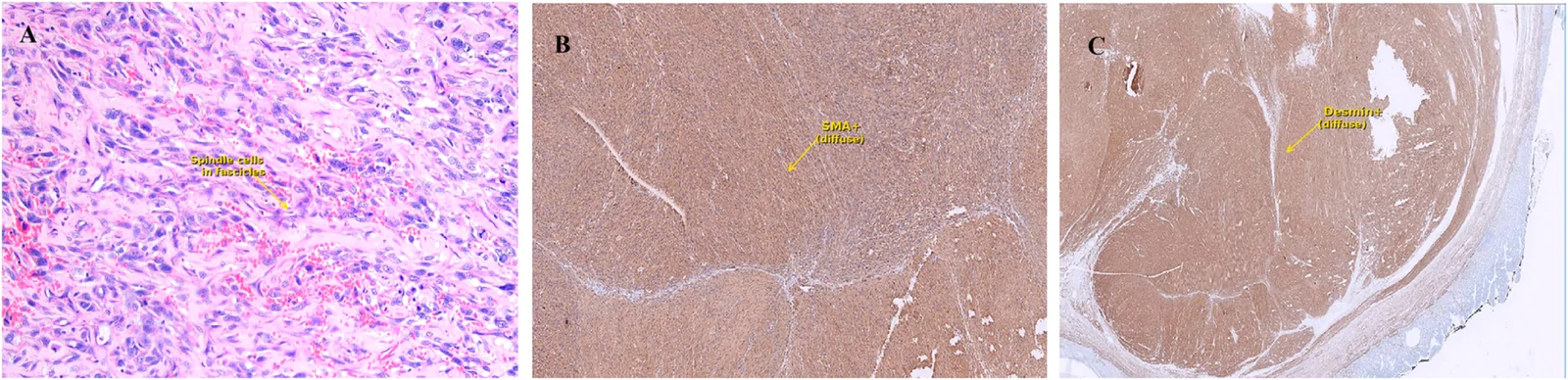
Microscopic image of the resected specimen. (A) H&E (400× magnification) showing spindle cells arranged in fascicles; (B) IHC panel: SMA+ (400× magnification); (C) IHC panel: desmin+ (400× magnification). H&E, hematoxylin and eosin; IHC, immunohistochemistry; SMA, smooth muscle actin.

**Table 1:** Timeline of clinical events.

Period	Event	Details
2 days before presentation (day: −2)	Symptom onset	Abdominal pain, abdominal distension, constipation, and vomiting
Day of presentation (day 0)	Physical examination	Tachycardic, normotensive, abdominal distension, and tenderness
	Laboratory tests	Mild leukocytosis
		Plain abdominal X-ray: multiple air-fluid levels in the center.
		CECT of the abdomen and pelvis: typical “target sign” consistent with jejuno-jejunal
		Intussusception with a 4.5 × 4 cm mass lesion serving as the lead point
Next day (day 1)	Exploratory laparotomy	Jejuno-jejunal intussusception with a soft tissue lesion as lead point located approximately 40 cm distal to the duodenojejunal flexure.
		Segmental resection with mesenteric lymphadenectomy and end-to-end anastomosis was done.
		Specimen was sent for histopathology.
Postoperative period	Recovery	Uneventful recovery. Discharged on day 7.
2 weeks after operation	Follow-up	MDT arranged.
		Adjuvant chemotherapy initiated.
6 months post-surgery	Follow-up	Patient is asymptomatic.
		CECT scan of the abdomen showed no residual growth, lymphadenopathy, or metastasis.
		Colonoscopy and upper GI endoscopy are normal.

## Discussion

Primary small bowel malignancies are rare compared with other sites of the GIT. Multiple hypotheses have been proposed to describe the causes of their rarity, including shorter transit time leading to decreased exposure to carcinogens and toxins, enzymes in the small intestine that reduce the production of free radicals, and surveillance by lymphoid tissues^[^15–17^]^. The histologic types of small bowel malignancies are (in decreasing order of frequency) adenocarcinoma, lymphoma, GIST, and sarcoma^[^5–7^]^.

Small-intestinal LMS, a malignant tumor of smooth muscle origin, is exceedingly rare and was historically confused with GIST until immunohistochemistry enabled its distinction^[^8,18^]^. Symptoms are nonspecific, and patients often present with advanced disease. Diagnosis of small-intestinal LMS is very challenging due to the rarity of the disease, nonspecific symptoms, and limitations of endoscopic methods^[^12^]^. Upper GI endoscopy and colonoscopy are not helpful. Advanced imaging, such as CT enterography, MR enterography, and capsule endoscopy, is effective^[^11^]^. Despite advances in imaging, preoperative differentiation of the nature of the tumor is challenging. Thus, histopathology and immunohistochemistry are the mainstays of disease confirmation, as noted in the present case. However, the morphological features of LMS and GIST are very similar; therefore, immunohistochemistry is essential for distinguishing between these two types^[^12^]^. GIST is the most common mesenchymal tumor of the GIT, arising from the interstitial cells of Cajal, and typically shows positivity for CD117, DOG-1, and S-100 on immunohistochemistry. In contrast, LMS originates from smooth muscle cells and shows positivity for SMA and desmin, and negativity for CD117, DOG-1, and S-100 on immunohistochemistry^[^12,16^]^. The immunohistochemical findings of the present case illustrate strong positivity for SMA and desmin and negativity for CD117, DOG-1, and S-100.

In the present case, several factors compounded the diagnostic difficulty. The lesion in the jejunum is inaccessible to both upper GI endoscopy and colonoscopy, precluding direct visualization and preoperative biopsy. CECT, while identifying the intussusception and lead-point mass, could not reliably differentiate the underlying tumor histology. Tumor markers (CEA, CA 19-9) were unremarkable, offering no adjunctive diagnostic value. Consequently, a definitive diagnosis was only possible postoperatively through histopathology and immunohistochemistry. This indicates that in cases of small bowel intussusception with an LP, preoperative diagnostic certainty is often unattainable, and surgical exploration with histopathological confirmation remains both diagnostic and therapeutic.

Intussusception may occur at different locations within the small and large intestines. There are four distinct types based on the LP: entero-enteric (involving only the small intestine), colo-colic, ileo-colic, and ileocecal^[^19^]^. In most adult cases, intussusception occurs in the small intestine with an LP, typically a benign condition such as adhesions, a diverticulum, inflammatory polyps, or benign tumors^[^3^]^. In rare circumstances, the LP may be malignant, most commonly metastatic (derived from melanoma, lung, or breast cancer), colonic adenocarcinoma, or lymphoma. Uncommon malignant triggers causing small-bowel intussusception include GIST, carcinoid tumors, neuroendocrine tumors, and LMS^[^20^]^. CT imaging remains the gold standard for preoperative diagnosis of intussusception, demonstrating the classic “target” or “sausage-shaped” mass^[^3^]^.

The clinical presentation of small-intestinal LMS as the LP of adult intussusception is a rare clinical scenario. Malignancy as an LP is more common in colonic intussusception (62.5%) than in small-intestinal intussusception (16.7%)^[^21^]^. While there are a handful of published cases of ileal LMS presenting with intussusception, cases involving the jejunum are even more sporadically reported^[^12,22,23^]^.

Complete surgical excision with clear margins is the mainstay of treatment^[^16^]^. The role of adjuvant therapy remains a matter of significant debate due to a lack of standardized guidelines. Because of the rarity of the disease, large-scale randomized controlled trials are lacking. Therefore, clinicians must adopt management strategies from soft tissue sarcoma protocols. In the present case, the multidisciplinary tumor board opted for adjuvant chemotherapy based on the tumor’s size (4.5 cm), mitotic activity (4–5/10 HPF), high tumor grade, Ki-67 index (70–80%), and the established poor prognosis of small-intestinal LMS.

The prognosis of small intestinal LMS depends on tumor size, mitotic index, and completeness of resection. Overall prognosis is very poor. The 5-year survival rate of patients with LMS of the small intestine ranges from 10% to 48%^[^5^]^. Our patient has remained in good condition during 6 months of follow-up. However, patients should be followed for a longer duration to draw definitive conclusions about long-term prognosis.

This case emphasizes the need for larger multi-institutional registries and pooled case-series analyses of jejunal LMS to generate disease-specific evidence. These could inform future revisions of soft tissue sarcoma guidelines to include site-specific recommendations for adjuvant therapy, surveillance intervals, and long-term follow-up in this rare tumor type.

This case has several strengths. It presents a rare “rare-on-rare” pathology – jejunal LMS as the LP of jejuno-jejunal intussusception – with complete clinical, radiological, and histopathological documentation, including a full immunohistochemical panel that definitively distinguishes LMS from GIST. The diagnostic and therapeutic pathway reflects genuine multidisciplinary collaboration, with the adjuvant chemotherapy decision made by a multidisciplinary tumor board. Complete surgical resection with clear margins and an uneventful postoperative course demonstrate a favorable short-term outcome. This case also has notable limitations. Most significantly, follow-up was limited to 6 months, which is insufficient to draw conclusions about long-term recurrence, metastasis, or survival. Longer follow-up is planned and will be necessary to determine the definitive prognosis. As a single case report, the findings are inherently non-generalizable. The adjuvant chemotherapy regimen was selected in the absence of disease-specific randomized trial evidence, an approach with an uncertain risk-benefit profile in this specific tumor subtype that could not be fully assessed within the scope of this report. No genetic or molecular profiling was performed, which might have added further diagnostic and prognostic insights. These limitations were addressed as far as possible by grounding management decisions in established multidisciplinary sarcoma protocols and by transparently reporting the limited follow-up duration rather than overstating prognostic conclusions.

## Conclusion

Jejunal leiomyosarcoma as the LP of jejuno-jejunal intussusception is an exceptionally rare entity. Adult intussusception requires evaluation for an underlying lesion, and immunohistochemistry is essential to distinguish LMS from GIST.

## Ethical approval

Ethical approval is exempt for the publication of anonymous case reports by the Ethical Review Committee of Chittagong Medical College, Chattogram, Bangladesh.

## Data Availability

This published article includes all the required data.
